# Comparison of long term efficacy and cost-effectiveness of omalizumab in 150 mg and 300 mg doses in patients with chronic spontaneous urticaria^☆^

**DOI:** 10.1016/j.abd.2024.02.006

**Published:** 2024-11-05

**Authors:** Fikriye Kalkan, Sait Yeşillik, Fevzi Demirel, Ezgi Sönmez, Yasemin Balaban, Mustafa İlker İnan, Özgür Kartal

**Affiliations:** Department of Immunology and Allergy, University of Health Sciences, Gulhane Training and Research Hospital, Ankara, Turkey

**Keywords:** Chronic urticaria, Omalizumab, Symptom assessment

## Abstract

**Background:**

Chronic spontaneous urticaria (CSU) is a clinical condition that affects patients quality of life. Omalizumab is preferred in antihistamines resistant CSU cases. Urticaria activity score-7 (UAS-7) is a scale that shows the severity of the disease.

**Objectives:**

The authors aimed to compare the long-term (60 months) efficacy and side effects of 150 mg and 300 mg doses of omalizumab in patients with CSU.

**Methods:**

108 patients followed up at the clinic with the diagnosis of CSU were included. Omalizumab was started in patients who were resistant to conventional CSU treatment. Two groups were formed to receive 150 mg and 300 mg doses of omalizumab. Urticaria activity score (UAS-7), antihistamine usage, time to achieve disease-free stage, relapse after treatment, and side effects of omalizumab treatment were compared in the two groups.

**Results:**

There were no statistically significant differences between the groups regarding basal characteristics and laboratory findings. Average follow-up time was sixty months. UAS-7 scores were similar in the follow-up. There were no adverse events in both groups.

**Study limitations:**

Retroactive design and single-center nature to reach a more significant number of patients. Lack of patients receiving the lowest dose 75 mg and the highest dose 600 mg of omalizumab. Absence of total body mass indexes of all patients. Besides, the use of distinct drugs may contribute to non confident results and is another limitation of this study.

**Conclusion:**

Since there is no significant difference between 150–300 mg omalizumab doses regarding long-term treatment efficacy and side effects in CSU patients, starting treatment with a 150 mg dose may be suitable. In patients who do not respond to 150 mg, the omalizumab dose can be increased to 300 mg. It will prevent unpredictable dose and time-dependent complications and will be a cost-effective approach even in strong economies.

## Introduction

Urticaria is characterized by itching, redness, the development of angioedema, or both. Clinical presentations lasting less than six weeks are referred to as acute urticaria, while clinical presentations lasting six weeks or longer are termed chronic urticaria.^1^ More than 1% of patients may still have symptoms for 5–10 years.^2^, ^3^ Approximately 60% and 30% of patients with moderate-to-severe disease still have symptoms after 2 and 5 years, respectively.^4^

One form of chronic urticaria is idiopathic urticaria, also known as Chronic Spontaneous Urticaria (CSU), but has no known external trigger, although an autoimmune basis has been widely investigated. Approximately two-thirds of patients with chronic urticaria are diagnosed with CSU.^5^

Antihistamine treatment is given in the treatment of urticaria. Antihistamine treatment can be increased up to four times depending on the control of the patient's complaints. Omalizumab, an anti-IgE antibody, is started in patients who do not respond to high-dose antihistamine treatment. Omalizumab is the only licensed biologic agent treatment for urticaria for patients who do not show sufficient benefit from treatment with a 2nd generation antihistamine. Omalizumab (anti-IgE) is very effective and safe in the treatment of CSU. In CSU, Omalizumab prevents healing and the development of angioedema, significantly improving quality of life.^1^, ^6^ Omalizumab is an effective treatment option for patients with moderate-to-severe symptoms of CSU and angioedema unresponsive to high-dose antihistamine therapy.^7^

Omalizumab is a recombinant humanized monoclonal antibody that reduces free IgE and functions by binding to the high-affinity receptor for the Fc region of IgE (FcεRI).^8^

The Urticaria Activity Score (UAS-7) score is filled in by the patient every day for seven days. It is proportional to the severity of the disease used to determine the severity of whealing and itching.^9^, ^10^

This study was planned to compare the Urticaria Activity Scores (UAS-7) before and after omalizumab treatment, the need for antihistamines, the patients who relapsed after the 60th month, and the recurrence and life-threatening adverse severe reactions in CSU patients according to 150 mg or 300 mg omalizumab doses.

## Materials and methods

Chronic Spontaneous Urticaria patients that had been unresponsive to high-dose antihistamine therapy for at least six months and subsequently received omalizumab treatment were included in the study. All patients included in the study did not receive adequate clinical response despite 4-fold doses of anti-H1 before starting omalizumab treatment. These patients were divided into two groups, receiving 150 mg and 300 mg of omalizumab, respectively. Omalizumab was applied every four weeks.^11^

Patients with chronic inflammatory conditions, those receiving anti-inflammatory treatment, individuals receiving immunosuppressive therapy for other medical conditions, cancer patients receiving alternative treatments, patients with psychiatric illnesses causing follow-up and treatment challenges, individuals undergoing immunosuppressive treatment for any reason, and those with a history of hypersensitivity reactions to omalizumab were excluded from the study.

The study included a comparison of the Urticaria Activity Score (UAS-7) before and after omalizumab treatment between the two groups, as well as an assessment of the use of antihistamines during omalizumab treatment, the occurrence of relapses after treatment, and the inquiry and comparison of severe life-threatening side effects associated with omalizumab therapy among the groups.

The Urticaria Activity Score (UAS-7) is a scoring system used by patients to assess the severity of their condition by recording the intensity of swelling and itching for seven days. In the UAS scale, the patient evaluates the severity of swelling and itching once daily for one week. UAS-7 values range from 0 to 42, with higher values indicating higher disease activity. In the UAS scale, the patient assesses the severity of swelling and itching once daily (every 24 hours). A UAS-7 score of 6 or below indicates reasonable control, 7–15 suggests mild activity in urticaria, 16–27 indicates moderate activity in urticaria, and 28–42 suggests severe activity in urticaria.^9^, ^12^

The use of high-dose antihistamines refers to increasing the dosage of second-generation antihistamines up to four times the standard dose.^1^ Regular antihistamine use means taking antihistamines every day. As-needed antihistamine treatment, on the other hand, entails using antihistamines only when symptoms are present.

The research protocol was approved by the Gulhane Education and Research Hospital Clinical Research Ethics Committee (E-50687469-799-2023/12). Since this study is retrospective, informed consent was not obtained. Patient data were accessed from the hospital automation system and patient records.

### Statistical analysis

Continuous variables are presented as mean ± standard deviation, while categorical variables are given as percentages. The Kolmogorov-Smirnov test was used to verify the normality of the distribution of continuous variables. Statistical analysis of clinical data between the two groups consisted of unpaired *t*-tests for parametric data, and Mann-Whitney *U*-test analysis for nonparametric data. Continuous variables and categorical variables were analyzed by the Chi-Squared statistic tests, Student's *t*-test, or Kruskal Wallis test when appropriate. Pearson’s coefficient was used as the association coefficient. Analyses were performed with PASW 22 (SPSS/IBM, Chicago, IL, USA) software, and a two-tailed p-value less than 0.05 was considered statistically significant.

## Results

The average age of the patients included in the study was 41.09 ± 12.4, and 60.2% were female. There were no statistically significant differences between the Omalizumab 150 mg and 300 mg dose groups regarding comorbidities, atopic diseases (asthma, allergic rhinitis, food sensitivities), autoimmune disease, baseline UAS-7 score averages, and laboratory values (p > 0.05). In both patient groups, all patients had been using high-dose antihistamines for at least six months (Table 1). Omalizumab 600 mg was not given to any of the patients.

**Table 1:** Baseline characteristics of omalizumab 150‒300 mg dose groups.

	**Total study population (n = 108)**	**Omalizumab 150 mg (n = 59)**	**Omalizumab 300 mg (n = 49)**	**p**
**Age, years**	41.09 ± 12,46	41.5 ± 13.4	40.5 ± 11.69	0.657
**Female gender, n (%)**	65 (60.2%)	31 (52.5%)	34 (69.4%)	0.076
**UAS-7 (basal)**	31.3 ± 6.89	31.75 ± 6.67	31.08 ± 7.25	0.760
**Angioedema, n (%)**	43 (32.3%)	20 (33.9%)	23 (46.9%)	0.168
**Atopic diseases, n (%)**	14 (13%)	8 (13.6%)	6 (12.2)	0.263
**Eosinophil, median (min‒max) 10^3^/mL**	0.161 (0‒0.6)	0.165(0‒0.5)	0.156 (0‒0.6)	0.780
**Total IgE kU/L, median (min‒max)**	259 (7–1168)	215 (7–1168)	376 (40–1056)	0.173
**Time since diagnosis for chronıc idiopatıc urtıcarıa month, (min‒max)**	31.62 (3–85)	34.50 (3–85)	28.14 (3–84)	0.643

UAS, Urticaria Activity Score; IgE, Immunoglobulin E.

The average UAS-7 scores of patients who received 150 mg and 300 mg of Omalizumab significantly decreased between the baseline UAS-7 score and the UAS-7 score in the 12th week (p < 0.001). In the 12th week, there was no statistically significant difference in UAS-7 values between patients receiving 150 mg and 300 mg of Omalizumab (p = 0.299). When examining the UAS-7 scores, it was observed that out of the 59 patients receiving 150 mg of Omalizumab, 52 (88.1%) achieved reasonable control, and 7 (11.9%) had mild urticaria activity according to UAS-7. One patient had moderate urticaria activity. It was observed that these seven patients were continuing to use antihistamines regularly. When assessing the UAS-7 scores, it was observed that out of the 49 patients receiving 300 mg of Omalizumab, 46 (93.9%) achieved reasonable control, while 3 (6.1%) still had mild urticaria activity according to UAS-7. It was also noted that these three patients continued to use antihistamine treatment regularly (Table 2).

**Table 2:** Comparison of omalizumab 150‒300 mg dose groups in terms of effectiveness.

	**Basal**		**12 week**	
	150 (n = 59)	300 (n = 49)	150 (n = 59)	300 (n = 49)
UAS-7 stage 1, n (%) (0–6)	0 (0%)	0 (0%)	52 (88.1%)	46 (93.9%)
UAS-7 stage 2, n (%) (7–15)	2 (3.4%)	1 (2%)	6 (10.2%)	3 (6.1%)
UAS-7 stage 3, n (%) (16–27)	15 (25.4)	15 (30.6%)	1 (1.7%)	0
UAS-7 stage 4, n (%) (28–42)	42 (71.2%)	33 (67.3%)	0	0
Antihistamine usage, n (%)				
None	0	0	34 (57.6%)	34 (69.4%)
Use when necessary	0	0	16 (27.1%)	11 (22.4%)
Using regularly	59 (100%)	49 (100%)	9 (15.3%)	4 (8.2%)

UAS, Urticaria Activity Score.

Out of the patients who initially received Omalizumab 150 mg, seven patients (11.9%) continued to use regular antihistamine treatment alongside Omalizumab 150 mg, but the clinical optimal improvement was not achieved, so they were switched to Omalizumab 300 mg. In their evaluation in the 24th week, it was observed that UAS-7 scores showed 5 of these patients had achieved reasonable control. The remaining two patients continued at a mild control level and continued to use antihistamines regularly.

Among the patients who initially received Omalizumab 300 mg, 3 of them had mild urticaria activity according to their UAS-7 score in the 12th week, and these patients continued to use antihistamines regularly alongside Omalizumab 300 mg. In their evaluation in the 24th week, it was seen that all 3 of these patients remained at a mild control level and continued to use antihistamines regularly.

For the rest of the patients receiving Omalizumab 150 mg and Omalizumab 300 mg, reasonable control continued at the 24th week.

The clinical characteristics of 7 patients who did not achieve reasonable control after receiving Omalizumab 150 mg in the 12th week and 3 patients who did not achieve reasonable control after receiving Omalizumab 300 mg in the 12th week are shown in Table 3.

**Table 3:** The clinical characteristics of patients who did not achieve reasonable control after receiving omalizumab.

	**P1**	**P2**	**P3**	**P4**	**P5**	**P6**	**P7**	**P8**	**P9**	**P10**
**Age, years**	39	43	53	62	51	50	41	33	48	44
**Gender, M‒F**	F	F	F	M	F	F	F	M	M	F
**Atopic disease**	–	–	–	–	+	–	+	–	–	–
**Angioedema**	–	–	+	–	–	+	–	–	+	–
**Time since diagnosis for chronıc idiopatıc urtıcarıa month) median**	82	23	13	20	46	24	10	5	24	10
**Total IgE kU/L**	0	233	1	264	191	96	1	5	1168	222
**Eosinophil, 10^3^/mL**	100	200	0	0	100	0	200	400	300	100
**Omalizumab dose, mg**	150	150	150	150	150	150	150	300	300	300
**Antihistamine use after starting omalizumab**	Yes	Yes	Yes	Yes	Yes	Yes	Yes	Yes	Yes	Yes
**Recurrence after 60 months**	Yes	Yes	No	No	No	No	No	No	No	No

P, Patient; M, Male; F, Female; UAS, Urticaria Activity Score; IgE, Immunoglobulin E.

It was observed that 36.5% of the patients receiving 150 mg of Omalizumab and 19.6% of the patients receiving 300 mg of Omalizumab experienced urticaria recurrence 60 months or more after treatment (p = 0.056) (Table 4). There were no statistically significant differences between the two groups regarding mild reactions such as injection site pain, swelling, itching, and redness. Additionally, no life-threatening serious adverse events were observed in either group.

**Table 4:** Treatment continuity at 5 years in patients receiving omalizumab 150‒300 mg doses.

		**150 mg (n = 52)**		**300 mg (n = 56)**		**p**
		**n**	**%**^a^	**n**	**%**^a^	
**Time**	Under 60-months	33	63.5	45	80.4	0.056^b^
	60-months and above	19	36.5	11	19.6	

a Column percentage.

b Pearson Chi-Square test.

## Discussion

In the present study, the authors showed that there was no significant difference in post-treatment evaluation between patient groups using 150 mg or 300 mg omalizumab doses in CSU patients in terms of UAS-7 scores, antihistamine need, relapses at 60 months and above, and serious adverse events.

CSU is a chronic skin condition that affects the quality of life and social relationships of patients. In its treatment, second-generation antihistamines are used as the first-line therapy. However, in cases where high-dose antihistamines are ineffective, omalizumab, a recombinant humanized monoclonal antibody, is employed.^13^ Patients should receive 150–300 mg of omalizumab every four weeks as a treatment dose.

In the new EAACI guidelines,1 omalizumab at 300 mg is recommended. However, this study is retrospective, so patients were initiated on either 150 mg or 300 mg of omalizumab by the previous guidelines.^11^

In studies comparing placebo and omalizumab treatment in patients with chronic spontaneous urticaria unresponsive to high-dose antihistamines, it has been observed that patients receiving omalizumab showed better results in terms of both the Chronic Urticaria Quality of Life Index and the time to disease recurrence. The adverse events identified in patients using omalizumab were consistent with a favorable safety profile.^14^, ^15^, ^16^

In another meta-analysis conducted by Zhao and colleagues in 2016, 7 studies were compared, involving 1312 patients with chronic urticaria, to assess the effectiveness of omalizumab compared to placebo in a controlled setting. In all the studies, it was observed that omalizumab treatment significantly reduced and controlled CSU symptoms compared to placebo. Patients treated with omalizumab had their symptoms better controlled than those on placebo. Furthermore, it was noted that omalizumab 300 mg was better tolerated than omalizumab 600 mg. It was observed that there was an improvement in the urticaria activity score in chronic urticaria patients receiving omalizumab 150 mg and omalizumab 300 mg compared to placebo in another study.^5^ Similarly, in the present study, it was observed that in patients who had not responded to high-dose antihistamine therapy for at least six months and whose symptoms were not controlled, omalizumab treatment provided effective control for most patients.

In another meta-analysis conducted by JIA HX and colleagues in 2020, 1612 patients were compared with 1251 patients in the placebo group. The study found that omalizumab was more effective in symptom control than placebo. It was noted that patients receiving omalizumab 600 mg had a higher incidence of adverse events.^17^ Publications show that the frequency of adverse events increases with the dose.^18^ During omalizumab treatment, side effects such as hypersensitivity reactions, injection-related reactions, acute asthma symptoms, eosinophilic diseases, fever, joint pain, rash, and others can be observed.^19^, ^20^

In this study, there was no significant difference in reactions such as pain, swelling, itching, and redness at the injection site between the two groups, and no serious adverse events were observed between the 150–300 mg two groups. In light of the above studies, the possibility of adverse events may increase as the dose increases. In this context, the authors think the initial low-dose strategy, which is routine in clinical practice for most diseases, should be preferred.

Marcus Maurer and colleagues studied, 323 symptomatic patients with chronic idiopathic urticaria who were followed to receive three subcutaneous injections of omalizumab or placebo at doses of 75 mg, 150 mg, or 300 mg, four weeks apart, followed by a 12-week observation period. It was found that omalizumab administered as three doses of 75 mg, 150 mg, or 300 mg at 4-week intervals significantly reduced symptoms compared to placebo. In patients receiving 150 mg or 300 mg omalizumab, a significant improvement in urticaria activity score was observed at the end of the 12th week compared to baseline.^8^

Similarly, in this study, no significant difference was observed in effectiveness between 150–300 mg doses in the evaluation at the end of the 12th week. However, omalizumab was switched to 300 mg in 7 patients whose complaints did not improve despite taking 150 mg. This suggests that only patients who do not respond to 150 mg can be switched to the upper dose of 300 mg.

In a study conducted by Saini et al., patients given 300 mg and 600 mg omalizumab doses were evaluated at the 12th week, and a significant improvement was observed between the two groups.^21^ In the ASTERIA I study, patients taking 300 mg and 150 mg were compared, and it was concluded that the 300 mg dose provided symptom control earlier than the 150 mg dose.^18^

Although it was observed in this study that symptom relief was shorter at the 300 mg dose, similar results in terms of recurrence were detected between 150–300 mg doses in the present study in the long-term follow-up. Of course, the medication must control the symptoms in a short time. However, long-term results are also a determining criterion in treatment continuity. The present study has a recurrence follow-up period of 60 months, which is longer than all three other studies.

Kim et al. started treatment with omalizumab 150 mg in 179 patients with chronic spontaneous urticaria, and at the end of the 12th week, complete control was achieved in 158 patients.^22^ Likewise, in this study, some patients were started on 150 mg omalizumab, while others were started on 300 mg omalizumab. No significant difference was observed between both groups.

Today, treatment costs are a severe problem worldwide. Achieving cost-effective treatment for patients is not only a financial problem but also a health problem regarding its results.^23^, ^24^, ^25^, ^26^, ^27^ In this context, 150 mg and 300 mg doses were compared in the present study, and no significant difference was found in treatment effectiveness. Based on this, a 150 mg dose, which may be more cost-effective than 300 mg, can be used in CSU patients. In the studied country omalizumab 150 mg flacon is available. When you prefer for initial dose of omalizumab 300 mg, costs increase two-fold. For this reason, it may be a better choice to start with 150 mg initially and then switch to 300 mg, depending on the evaluation at the end of the 12th week.

## Conclusion

Since there is no significant difference between 150–300 mg omalizumab doses regarding long-term treatment effectiveness and side effects in CSU patients, it may be appropriate to start with 150 mg as the treatment dose. Treatment with a low dose will be more cost-effective. It may be reasonable to start with omalizumab 150 mg first and switch to omalizumab 300 mg if no clinical improvement is achieved in subsequent controls.

## Limitation

The primary limitations of the present study include its retroactive design and single-center nature in reaching a more significant number of patients. Additionally, the need for patients receiving the lower dose of omalizumab 75 mg and the highest dose of omalizumab 600 mg can be considered a shortcoming of the study. Furthermore, the lack of total body mass indexes of all patients divided into different omalizumab dose groups is a limitation. Also, the use of distinct drugs may contribute to no confident results, which is another limitation of this study. Finally, the number of patients is small, and studies can be conducted with more patients. Of course, more comprehensive, prospective studies are needed.

## Financial support

None declared.

## Authors’ contributions

Fikriye Kalkan: The study concept and design, data collection, or analysis and interpretation of data, writing of the manuscript or critical review of important intellectual content, data collection, analysis and interpretation, effective participation in the research guidance, intellectual participation in the propaedeutic and/or therapeutic conduct of the studied cases, critical review of the literature and final approval of the final version of the manuscript.

Sait Yeşillik: The study concept and design, data collection, or analysis and interpretation of data, effective participation in the research guidance and final approval of the final version of the manuscript.

Fevzi Demirel: Data collection, or analysis and interpretation of data, statistical analysis, data collection, analysis and interpretation, effective participation in the research guidance, intellectual participation in the propaedeutic and/or therapeutic conduct of the studied cases.

Ezgi Sönmez: Data collection, analysis and interpretation, effective participation in the research guidance, intellectual participation in the propaedeutic and/or therapeutic conduct of the studied cases.

Yasemin Balaban: Data collection, analysis and interpretation, effective participation in the research guidance, intellectual participation in the propaedeutic and/or therapeutic conduct of the studied cases.

Mustafa İlker İnan: Data collection, analysis and interpretation, effective participation in the research guidance, intellectual participation in the propaedeutic and/or therapeutic conduct of the studied cases.

Özgür Kartal: Data collection, or analysis and interpretation of data, statistical analysis, writing of the manuscript or critical review of important intellectual content, effective participation in the research guidance, intellectual participation in the propaedeutic and/or therapeutic conduct of the studied cases, critical review of the literature and final approval of the final version of the manuscript.

## Conflicts of interest

None declared.
